# Subarachnoid hemorrhage: who dies, and why?

**DOI:** 10.1186/s13054-015-1036-0

**Published:** 2015-08-31

**Authors:** Hector Lantigua, Santiago Ortega-Gutierrez, J. Michael Schmidt, Kiwon Lee, Neeraj Badjatia, Sachin Agarwal, Jan Claassen, E. Sander Connolly, Stephan A. Mayer

**Affiliations:** Department of Neurology, Columbia University College of Physicians and Surgeons, 177 Fort Washington Ave, New York, NY 10032 USA; Department of Neurology, University of Iowa, 200 Hawkins Drive, Iowa City, IA 52242 USA; The University of Texas at Houston, 6431 Fannin St., Houston, TX 77030 USA; The University of Maryland, 22 South Greene St., Baltimore, MD 21201 USA; Department of Neurosurgery, Columbia University College of Physicians and Surgeons, 710 West 168th St., New York, NY 10032 USA; Icahn School of Medicine at Mount Sinai, One Gustave L. Levy Place, Box 1522, New York, NY 10029-6574 USA

## Abstract

**Introduction:**

Subarachnoid hemorrhage (SAH) is a devastating form of stroke. Causes and mechanisms of in-hospital death after SAH in the modern era of neurocritical care remain incompletely understood.

**Methods:**

We studied 1200 consecutive SAH patients prospectively enrolled in the Columbia University SAH Outcomes Project between July 1996 and January 2009. Analysis was performed to identify predictors of in-hospital mortality.

**Results:**

In-hospital mortality was 18 % (216/1200): 3 % for Hunt-Hess grade 1 or 2, 9 % for grade 3, 24 % for grade 4, and 71 % for grade 5. The most common adjudicated primary causes of death or neurological devastation leading to withdrawal of support were direct effects of the primary hemorrhage (55 %), aneurysm rebleeding (17 %), and medical complications (15 %). Among those who died, brain death was declared in 42 %, 50 % were do-not-resuscitate at the time of cardiac death (86 % of whom had life support actively withdrawn), and 8 % died despite full support. Admission predictors of mortality were age, loss of consciousness at ictus, admission Glasgow Coma Scale score, large aneurysm size, Acute Physiology and Chronic Health Evaluation II (APACHE II) physiologic subscore, and Modified Fisher Scale score. Hospital complications that further increased the risk of dying in multivariable analysis included rebleeding, global cerebral edema, hypernatremia, clinical signs of brain stem herniation, hypotension of less than 90 mm Hg treated with pressors, pulmonary edema, myocardial ischemia, and hepatic failure. Delayed cerebral ischemia, defined as deterioration or infarction from vasospasm, did not predict mortality.

**Conclusion:**

Strategies directed toward minimizing early brain injury and aneurysm rebleeding, along with prevention and treatment of medical complication, hold the best promise for further reducing mortality after SAH.

**Electronic supplementary material:**

The online version of this article (doi:10.1186/s13054-015-1036-0) contains supplementary material, which is available to authorized users.

## Introduction

Subarachnoid hemorrhage (SAH) is devastating acute neurological disease that affects over 30,000 people every year in the United States [1–4]. Despite advances in medical and surgical management, SAH remains a major cause of premature mortality, accounting for 27 % of all stroke-related potential years of life lost before the age of 65 [5]. In a 1985 study, it was reported that SAH carried a 43 % risk of death immediately after ictus and a 57 % mortality rate at 6 months [6]. A systematic review in 1997 evaluated cases-fatality rates from 1960 to 1992 and found a 0.9 % decrease per year [7].

Well-established risk factors for mortality included poor clinical grade at presentation, older age, aneurysm rebleeding, large aneurysm size, and cerebral infarction from vasospasm [8]. The International Cooperative Aneurysm Study, conducted in the 1980s, pointed to vasospasm, direct effects of the primary hemorrhage, and rebleeding as the most frequent causes of mortality after SAH [9]. More recently, global cerebral edema, intraventricular hemorrhage, and medical complications have been identified as contributors to poor outcome after SAH [10–13]. In this study, our goal was to re-evaluate the causes and mechanisms of in-hospital mortality after SAH in a large contemporary single-center cohort.

## Methods

### Study population

All spontaneous SAH patients admitted to the Neurological Intensive Care Unit at Columbia University Medical Center between July 1996 and January 2009 were offered enrollment in the Columbia University SAH Outcomes Project. Both patients with and those without a documented aneurysm were included in the analysis. The study was approved by the Columbia University Institutional Review Board, and in all cases written informed consent was obtained from the patient or a legally authorized representative. Patients were admitted to a dedicated neurological intensive care unit (ICU) and given treatment according to a standardized management protocol that has been described in detail previously; see Methods Supplement (Additional file 1) for details regarding inclusion and exclusion criteria, aneurysm management, and a description of our ICU management protocol and how it evolved over time [12, 14, 15].

### Clinical and radiological assessment

Demographic data, social and medical history, and clinical features at onset were obtained shortly after admission. Neurological status was assessed with the Glasgow Coma Scale (GCS) [16] and the Hunt-Hess scale [17]. Physiologic stress was captured by using the Acute Physiology and Chronic Health Evaluation II (APACHE II) scale by calculating a physiological subscore after subtracting the GCS, age, and chronic health contributions to the total score [14]. Admission computed tomography (CT) scans, and those with significant interval changes during hospitalization, were evaluated by using the Modified Fisher Scale [15], Hijdra SAH sum score [18], and Intraventricular Hemorrhage Score [19] and for the presence of global cerebral edema [12]. During weekly meetings, a review of the entire hospital course was conducted to document and adjudicate important procedures, events, and complications according pre-specified criteria [10]. Delayed cerebral ischemia (DCI) was defined as neurological deterioration, cerebral infarction, or both, when the cause was felt to be vasospasm after careful exclusion of other causes [20].

### Outcome assessment

In-hospital mortality was used as the primary outcome for this analysis to allow for uniform evaluation of the causes, mode (i.e., brain versus cardiac), and level of support related to the dying process. The principal mechanism of death or neurological devastation leading to withdrawal of care was identified by adjudication of the study and clinical team after review of all pertinent clinical and radiographic findings. Primary causes of death were divided into eight categories based on the underlying pathophysiological mechanism: (1) direct effect of the primary hemorrhage, (2) aneurysm rebleeding, (3) cerebral infarction from vasospasm, (4) refractory cerebral edema leading to brain stem herniation, (5) hydrocephalus, (6) operative complications, (7) medical complications (e.g., fatal arrhythmia, pulmonary embolism, or multisystem organ failure who initially had a good neurological prognosis), and (8) other. Although different mechanisms could overlap in the same patient, only the adjudicated primary cause of death was considered.

### Witholding or withdrawal of support

Do-not-resuscitate (DNR) status was systemically documented when instituted. Withdrawal of supportive care was defined as the cessation of intensive care support such as mechanical ventilation and the beginning of comfort care [21]. The treating team actively withdrew life support only at the direction of family members on the basis of dismal expected prospects for recovery and the known wishes of the patient on the basis of previous written or verbal statements.

### Statistical analysis

Continuous variables were dichotomized to relevant clinical cut-points. Continuous variables were assessed for normality by using the Kolmogorov-Smirnov test. Normally distributed data were reported as a mean ± standard deviation, and non-parametric data were reported as median and interquartile range (IQR). Univariable associations were tested by using chi-squared or Fisher’s exact test for categorical variables, two-tailed *t* test for normally distributed continuous variables, and Mann–Whitney *U* test for non-normally distributed continuous variables. An initial multivariable analysis using logistic regression was performed to determine the relationship between premorbid demographic and admission clinical and radiographic variables and in-hospital mortality. For clinically intercorrelated variables that measure the same construct (e.g., admission clinical deficit measured by Hunt-Hess grade and GCS score), we selected the variable with the highest measure of association (odds ratio) and smallest *P* value to be included in the final model. After construction of the baseline model for prediction of mortality, adjusted odds ratios for specific hospital complications were calculated by adding each of these factors individually to the baseline model to evaluate their unique contribution. Receiver operating characteristic (ROC) analysis was used to assess the ability of various models to predict in-hospital mortality. Nagelkerke’s R-squared was used to estimate the percentage of the variance predicted by the combination of variables into the predicted model. Finally, a Hosmer-Lemeshow test was used to test goodness-of-fit of the each model. Residual statistics analysis was performed to evaluate the influence of isolated points into the model. Potential multicollinearity between the parameters of the final regression model was performed by calculating tolerance and variance inflation factor (VIF) coefficients. Significance was set at 0.05 for all analyses. All analyses were performed with commercially available statistical software (SPSS version 18.0; SPSS Inc., now part of IBM Corporation, Armonk, NY, USA).

## Results

### Overall mortality and mode of death

**Fig. 1:**
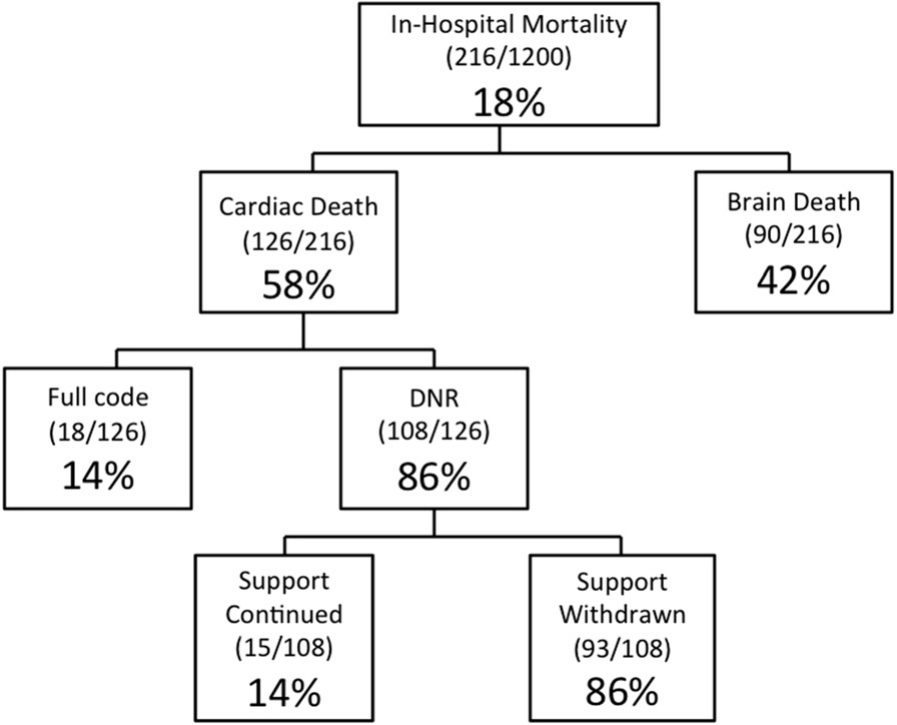
Flow diagram of in-hospital mortality after subarachnoid hemorrhage. *DNR* do-not-resuscitate

Of the 1200 patients enrolled between July 1996 and January 2009, 216 (18 %) died during hospitalization. Of those who died, 42 % succumbed to brain death. Of those who died of cardiac death, 86 % had a DNR order and 74 % had life support actively withheld or withdrawn (Fig. 1). Mortality according to admission Hunt-Hess clinical grade is displayed in Table 1. Fifty-seven percent of patients underwent an aneurysm clipping procedure (*N* = 687), 19 % (*N* = 231) were coiled, and 24 % underwent no procedure, due to either absence of an identifiable aneurysm (*N* = 148) or extremely poor clinical grade (*N* = 134). Mortality was higher among coiled (14 %) than clipped (7 %) patients. Median hospital lengths of stay were 14 (IQR 10–22) days for survivors and 5.5 (IQR 2–12) days among non-survivors (*P* = 0.001). Patients admitted in Hunt-Hess grade 3 to 5 condition represented over 90 % of those who died.

**Table 1:** Mortality according to admission Hunt-Hess grade

Hunt-Hess grade	Dead/Total, number	Proportion of study population, %	Mortality rate, %
1. Mild headache	12/342	19.5	3.5
2. Severe headache or cranial nerve deficit	6/186	15.5	3.2
3. Confusion, lethargy, or lateralized weakness	30/319	26.6	9.4
4. Stupor	41/173	14.4	23.6
5. Coma	127/180	15.0	70.5
Total	216/1200	100.0	18.0

### Timing of death

**Fig. 2:**
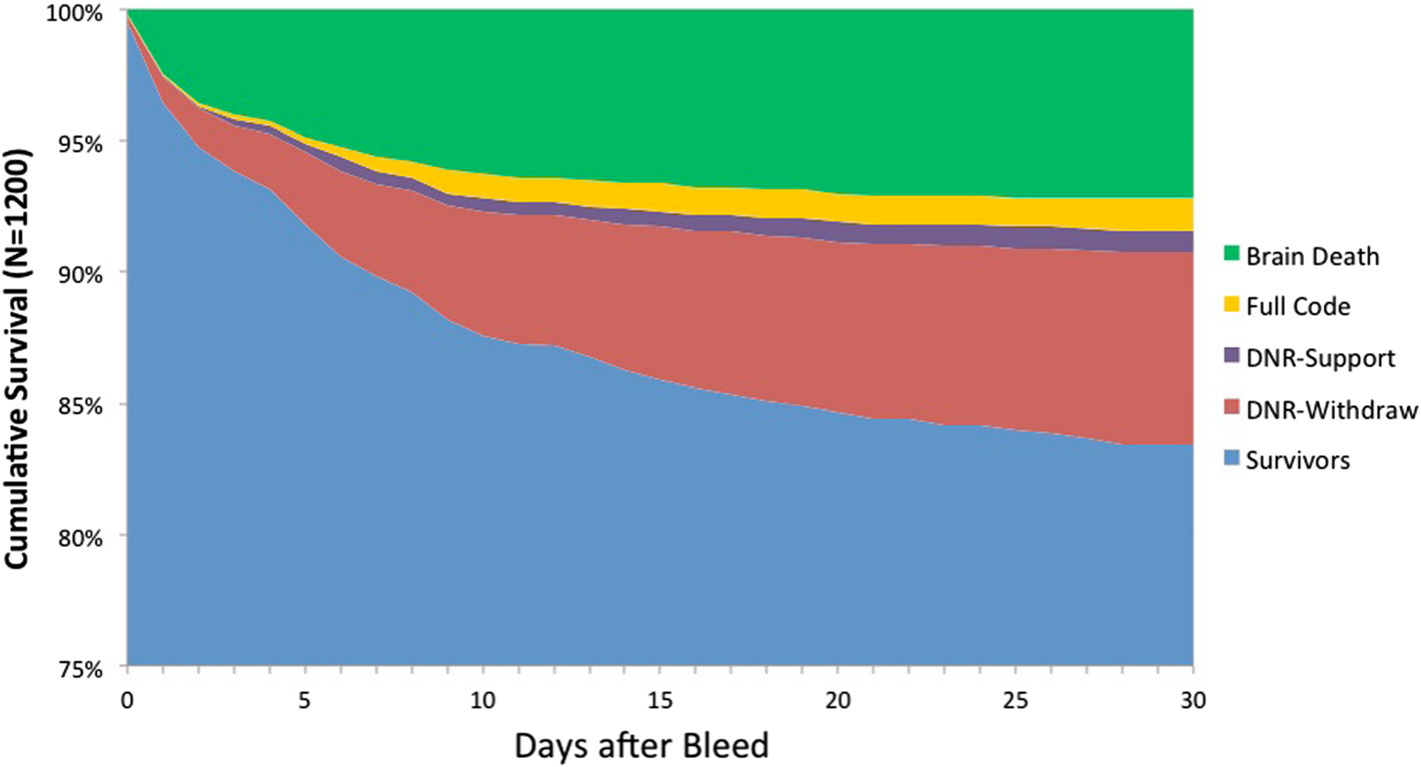
Survival analysis stratified by mode of death and level of support during the first 2 weeks after SAH. An additional 17 patients (8 % of those who died overall) died after SAH day 30 but prior to discharge. *DNR* do-not-resuscitate, *SAH* subarachnoid hemorrhage

**Fig. 3:**
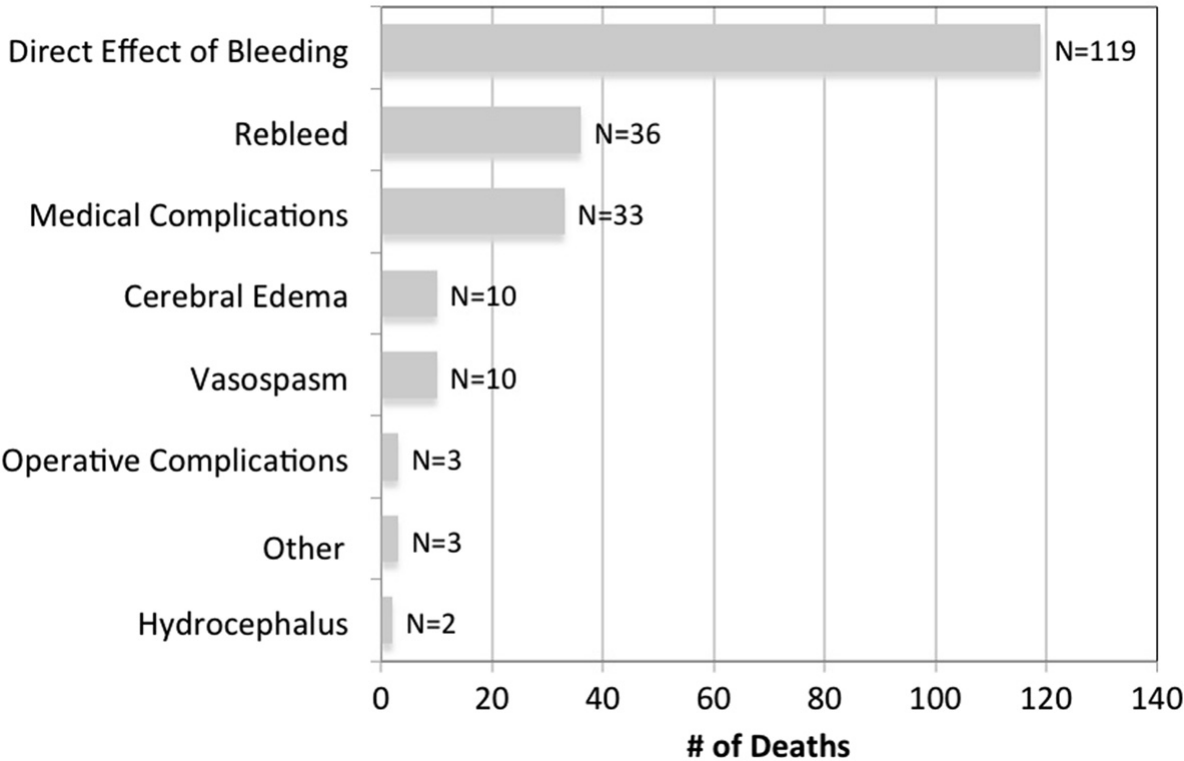
Adjudicated causes of death or neurological devastation leading to withdrawal of support. “Other” causes included prolonged coma after refractory status epilepticus, internal carotid artery rupture due to balloon angioplasty, and hemorrhagic conversion of infarct

Survival analysis stratified by mode of death and level of support is shown in Fig. 2. Thirty percent of deaths occurred within 48 h of admission, 56 % had occurred by SAH day 7, and 76 % had occurred by SAH day 14. The majority of brain dead patients (74 %, 67/90) died within 7 days of SAH, whereas death due to active withdrawal of support was more likely to occur after day 7 (55 %, 51/93). Overall, the most common adjudicated primary causes of death (Fig. 3) were direct effect of the primary hemorrhage (55 %), aneurysm rebleeding (17 %), medical complications (15 %), cerebral edema (5 %), and DCI from vasospasm (5 %).

### Temporal mortality trend

**Fig. 4:**
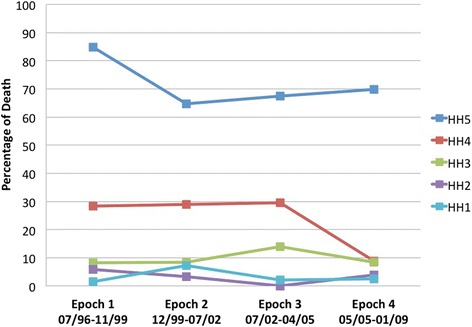
Hospital mortality according to admission Hunt-Hess grade over the 12.5-year study period. Each time epoch represents 300 consecutive admissions. A dramatic fall in mortality was observed among grade 5 patients between epochs 1 and 2; a similar reduction occurred among grade 4 patients between epochs 3 and 4. *HH* Hunt-Hess

Analysis of mortality over time according to admission clinical grade was performed by dividing the study population into four groups of 300 consecutively treated patients who received treatment over the course of about 3 years. Mortality remained consistently low for good-grade SAH patients (Hunt-Hess 1–3) over the 12-year period (Fig. 4), but there was a 20 % absolute reduction in mortality among grade 5 patients between the first and second time epochs (*P* = 0.046, χ^2^ test for epoch 1 versus epochs 2–4) and a similar 20 % reduction among grade 4 patients between the third and fourth time epochs (*P* = 0.007, χ^2^ test for epochs 1–3 versus epoch 4). The proportion of patients who underwent withdrawal of support, and the timing of withdrawal, did not change across time epochs.

### Admission predictors of mortality

Twenty-two admission variables related to demographics, medical history, clinical features, imaging findings, and physiology were associated with in-hospital mortality in univariate analysis (Table 2). In a multivariable logistic regression analysis, age, loss of consciousness at ictus, APACHE II physiological subscore, admission GCS score, aneurysm size, and Modified Fisher Scale at admission were found to be the most important independent admission risk factors associated with in-hospital mortality (Nagelkerke R-squared = 0.497; AUC = 0.90, 95 % CI 0.87–0.93, *P* < 0.0001) (Table 3). No interactions or collinearity was found between any of the predictors included in the final model. Hosmer and Lemeshow test indicated adequacy of model fitting (*P* = 0.116). Both Cook’s distance and Leverage residual coefficients were less than 1 for all individuals included in the study. Less than 5 % of all standardized residuals were outside ± 1.96.

**Table 2:** Baseline characteristics related to in-hospital mortality

	Entire cohort	Survivors	Non-survivors	
	*N* = 1200	*N* = 984	*N* = 216	*P*
Demographics				
Age	55 ± 15	53 ± 14	60 ± 16	0.000
Female gender	807 (67)	661 (67)	146 (68)	0.906
Non-white	601 (50)	491 (50)	110 (51)	0.903
Medical history				
Hypertension	542 (45)	421 (43)	121 (56)	0.000
Diabetes	89 (7)	76 (8)	13 (6)	0.916
Cigarette use in the last 6 months	669 (56)	577 (59)	91 (42)	0.690
Alcohol use in the last 6 months	652 (54)	587 (60)	65 (30)	0.000
Coronary artery disease	52 (4)	34 (4)	18 (8)	0.000
Chronic obstructive pulmonary disease	61 (5)	44 (5)	17 (8)	0.004
Admission clinical features				
Loss of consciousness at ictus	458 (38)	307 (31)	151 (70)	0.000
Tonic-clonic activity at ictus	130 (11)	96 (10)	34 (16)	0.002
Sentinel headache	203 (17)	162 (17)	41 (20)	0.032
Admission Glasgow Coma Scale score	14 (8–15)	15 (12–15)	5 (3–9)	0.000
Admission Hunt-Hess grade				0.000
1. Mild headache	342 (29)	330 (34)	12 (6)	
2. Severe headache	186 (16)	180 (18)	6 (3)	
3. Lethargy or confusion	319 (27)	289 (29)	30 (14)	
4. Stupor	173 (14)	132 (13)	41 (19)	
5. Coma	180 (15)	53 (5)	127 (59)	
Admission imaging findings				
Modified Fisher Score				0.000
0. No blood	66 (6)	61 (6)	5 (2)	
1. Focal or diffuse thin SAH	337 (28)	320 (33)	17 (8)	
2. Focal or diffuse thin SAH with bilateral IVH	106 (9)	82 (8)	24 (11)	
3. Focal or diffuse thick SAH	415 (35)	346 (35)	69 (32)	
4. Focal or diffuse thick SAH with bilateral IVH	246 (21)	148 (15)	98 (45)	
SAH sum score^a^	15 (7–21)	13 (6–20)	20 (15–25)	0.000
IVH sum score^b^	1 (0–4)	0 (0–3)	4 (1–9)	0.000
ICH present	199 (17)	126 (13)	73 (34)	0.000
Global cerebral edema	210 (18)	137 (14)	73 (34)	0.000
Hydrocephalus (bicaudate index >0.2)	350 (29)	271 (28)	79 (37)	0.000
Aneurysm characteristics				0.000
Anterior cerebral artery	318 (26)	275 (28)	43 (20)	
Internal carotid artery (includes P-comm)	322 (27)	277 (28)	45 (21)	
Middle cerebral artery	170 (14)	142 (14)	28 (13)	
Vertebrobasilar system	183 (15)	153 (16)	30 (14)	
No aneurysm identified	140 (12)	134 (14)	6 (3)	
No angiogram performed	67 (6)	3 (0.3)	64 (30)	
Aneurysm diameter, mm	7 [5–10]	7 [5–10]	10 (6–15)	0.000
Multiple aneurysms	295 (25)	256 (26)	39 (18)	0.954
Admission physiology				
Systolic blood pressure, mm Hg	156 [132–180]	154 [132–178]	164 (131–196)	0.003
Glucose level, mg/dl	142 [118–176]	136 [115–165]	180 [139–241]	0.000
Troponin level, ng/dl	0.26 [0.02–0.3]	0.1 [0.02–0.3]	0.3 [0.02–1.6]	0.000
APACHE physiological subscore^c^	5 [3–8]	5 [3–7)]	9 [6–12]	0.000

Values are presented as mean ± standard deviation, median [interquartile range], or number (percentage within each column).

*SAH* subarachnoid hemorrhage, *IVH* intraventricular hemorrhage, *ICH* intracerebral hemorrhage, *APACHE* Acute Physiology and Chronic Health Evaluation

^a^Scale 0 = no blood, 30 = complete filling of all cisterns and fissures

^b^Scale 0 = no IVH, 12 = complete filling of all ventricles

^c^Scale 0 = no physiological derangement, 44 = maximal physiological derangeme

**Table 3:** Admission predictors of in-hospital mortality: multivariable analysis

Variable	OR	95 % CI	*P* value
Age, years	1.04	1.0–1.1	0.00
Loss of consciousness at ictus	1.99	1.3–3.2	0.03
Admission GCS score	0.77	0.7–0.8	0.00
Aneurysm size, per mm	1.10	1.1–1.2	0.00
APACHE II physiologic subscore	1.08	1.0–1.1	0.02
Modified Fisher Scale score	1.25	1.0–1.5	0.03

*OR* odds ratio, *CI* confidence interval, *GCS* Glasgow Coma Scale, *APACHE II* Acute Physiology and Chronic Health Evaluation II

### Medical and neurological complications

The vast majority of medical complications were over-represented among patients who died (Table 4). After admission predictors of mortality were controlled for, complications that remained significantly associated with mortality included global cerebral edema, intracranial pressure (ICP) crisis or herniation events treated with bolus osmotherapy, hypotension (systolic blood pressure of less than 90 mm Hg) treated with pressors, congestive heart failure, aneurysm rebleeding, myocardial injury, and hepatic injury (Table 4). Of the 20 % of the study population (*N* = 239) with diagnosed DCI, 92 % received treatment with vasopressors and 38 % with endovascular therapy in the form of intra-arterial verapamil (89 %) or balloon angioplasty (45 %) or both. These proportions did not change significantly over the four time epochs. DCI did not predict mortality.

**Table 4:** Relationship of medical and neurological complications to in-hospital mortality

			Univariate/Unadjusted			Multivariate/Adjusted^a^		
	Survivors	Non-survivors	OR	95 % CI	*P* value	OR	95 % CI	*P* value
Fever >101.5 F	480 (49)	137 (63)	1.9	1.4–2.6	0.000	0.8	0.5–1.3	0.35
Hyperglycemia, >200 mg/dl	408 (42)	143 (66)	2.9	2.1–4.0	0.000	1.1	0.7–1.8	0.76
Hydrocephalus requiring EVD or VPS	289 (29)	142 (66)	4.7	3.5–6.5	0.417	1.4	0.9–2.2	0.15
Anemia requiring transfusion	319 (32)	75 (35)	1.1	0.8–1.6	0.000	0.7	0.45–1.0	0.10
Global cerebral edema	205 (21)	107 (50)	3.7	2.8–5.2	0.000	1.8	1.1–2.9	0.02
New infarct on CT scan	233 (24)	78 (36)	2.0	1.5–2.8	0.000	0.7	0.4–1.1	0.09
ICP crisis or herniation^a^	153 (16)	106 (49)	5.7	4.0–7.9	0.000	2.5	1.4–3.7	0.00
Hypotension, <90 mm Hg^b^	160 (16)	122 (57)	6.7	4.9–9.2	0.000	3.4	2.2–5.3	0.00
Pneumonia	191 (19)	67 (31)	1.9	1.4–2.7	0.000	0.6	0.4–1.0	0.06
Hypernatremia, >150 mEq/l	151 (15)	99 (46)	5.1	3.6–7.1	0.000	2.1	1.3–3.4	0.00
Urinary tract infection	226 (23)	13 (6)	0.2	0.1–0.4	0.881	0.1	0.0–0.2	0.00
Clinical deterioration from vasospasm	170 (17)	36 (17)	0.9	0.7–1.4	0.000	0.9	0.6–1.6	0.81
Pulmonary edema	137 (14)	68 (32)	2.9	2.0–4.1	0.000	1.3	0.8–2.0	0.33
Herniation	62 (6)	106 (50)	15.3	10.5–22.1	0.052	8.3	4.9–14.3	0.00
Hyponatremia, <130 mEq/l	140 (14)	20 (9)	0.6	0.4–1.0	0.010	0.5	0.3–0.96	0.04
Sepsis/Bacteremia	91 (9)	32 (15)	1.8	1.1–2.7	0.000	1.4	0.79–2.4	0.26
Arrhythmia	71 (7)	49 (23)	3.7	2.5–5.6	0.000	1.6	0.9–2.8	0.09
Aneurysm rebleeding	54 (6)	64 (30)	6.9	4.6–10.4	0.000	3.5	1.9–5.9	0.00
Congestive heart failure	62 (6)	39 (18)	3.3	2.1–5.1	0.001	2.2	1.3–3.8	0.00
Seizures	55 (6)	25 (12)	2.3	1.4–3.8	0.068	1.6	0.8–3.0	0.20
Delayed cerebral ischemia								
Symptomatic vasospasm without infarct	104 (11)	14 (7)	0.6	0.3–1.0	0.095	0.5	0.3–1.0	0.06
Symptomatic vasospasm with infarct	64 (7)	21 (10)	1.5	0.9–2.6	0.004	1.9	1.0–3.7	0.07
No symptomatic vasospasm with infarct	23 (2)	13 (6)	2.7	1.3–5.4	0.000	2.2	0.8–5.8	0.13
Non-neurogenic myocardial ischemia	46 (5)	43 (20)	5.1	3.3–8.1	0.000	2.8	1.6–5.1	0.00
Hepatic injury, AST or ALT >200 mg/dl	24 (2)	22 (10)	4.6	2.5–8.4	0.000	2.5	1.2–5.3	0.01
GI bleeding requiring transfusion	19 (2)	14 (7)	3.5	1.7–7.1	0.000	1.7	0.7–4.3	0.27

Values are presented as number (percentage)

*OR* odds ratio, *CI* confidence interval, *EVD* external ventricular drain, *VPS* ventriculoperitoneal shunt, *CT* computed tomography, *ICP* intracranial pressure, *AST* aspartate transaminase, *ALT* alanine transaminase, GI gastrointestinal

Adjusted ORs for in-hospital mortality are calculated individually for each complication after adjustment for admission mortality predictors (Table 3). See reference [9] for complete definitions

^a^Treated with osmotherapy

^b^Treated with vasopressors

## Discussion

In this contemporary single-center study of 1200 cases of SAH, hospital mortality was 18 %, which is on the low end of the range of 20–50 % previously reported in the literature [1, 3–8, 22, 23]. Although referral bias favoring transfer of good-grade patients can influence SAH mortality rates at tertiary care centers [24], Hunt-Hess grades in our study population were broadly represented and consistent with those found in epidemiologic studies (Table 1) [4–7]. More likely, our results reflect the current trend of improvement in SAH outcomes that has occurred over the past two decades, with more aggressive aneurysm treatment protocols and advanced critical care strategies directed at minimizing secondary injury [3, 7, 8]. This view is supported by the fact that mortality fell from approximately 85 % to 70 % among grade 5 patients early in the 12-year study period and from 30 % to 10 % among grade 4 patients near the end of the study period (Fig. 4). SAH mortality has consistently been shown to be lower at high-volume as opposed to low-volume centers [3, 25, 26]. As regionalization of complex stroke care at comprehensive stroke centers becomes more prevalent, risk-adjusted SAH mortality rates from specialized centers such as ours may serve as useful targets and benchmarks for evaluating and comparing quality of care between centers.

Forty-two percent of those who died in our study were pronounced brain dead. Most of these patients presented with loss of brainstem reflexes, had evidence of severe brain stem or diffuse cortical injury on CT, did not undergo angiography, and died quickly. Of the remaining patients, 50 % were DNR (86 % of whom had life support actively withdrawn) and only 8 % died despite full medical support (Fig. 1). Two recent single-center studies of in-hospital mortality after SAH reported similar rates of overall mortality (18–20 %) and withholding or withdrawal of support (50–76 %) compared with our study [22, 23]. In general, we withdrew support from poor-grade patients at our center after an initial trial of aggressive surgical and critical care intervention. This protocol involved aneurysm repair (with a bias toward coiling) and external ventricular drainage whenever feasible and has come to include continuous electroencephalography, invasive brain multimodality monitoring, hypothermia for refractory intracranial pressure, and intra-arterial verapamil, balloon angioplasty, and intrathecal nicardipine for vasospasm as our practice has evolved [8].

SAH is a complicated disease that can involve multiple types of neurological injury and systemic organ dysfunction; it may be impossible to identify the precise contribution of each individual process to a patient’s death. While acknowledging this problem, we sought to identify the “primary cause of death or neurological devastation leading to withdrawal of support” in weekly meetings of the research team after thorough review of all pertinent clinical and imaging data. The three most common adjudicated causes of death (Fig. 3) were direct effects of the primary hemorrhage (55 %), aneurysm rebleeding (17 %), and medical complications (15 %). These findings suggest that, as of today, little has changed since a 1994 population-based study from the Greater Cincinnati region reported that initial and recurrent aneurysm rebleeding are the major causes of death after SAH [27].

Multivariable analysis of admission predictors (Table 3) and delayed complications confirms (Table 4) our adjudicated causes of death. Admission GCS score and Modified Fisher Scale are robust markers of the severity of the initial bleeding event, and both predicted in-hospital mortality. Loss of consciousness at ictus is felt to reflect transient intracranial circulatory arrest, has been previously been linked to poor outcome and global cerebral edema at SAH onset [8, 28], and was also associated with mortality in our study. Global cerebral edema, a marker of diffuse brain injury that is usually present on admission CT, was associated with mortality when analyzed as a complication of SAH. Admission APACHE II physiological subscores capture extremes of blood pressure, hypoxia, fever, and other signs of systemic inflammation; their association with mortality suggests that these derangements may aggravate early brain injury [14]. Multivariable analysis confirmed aneurysm rebleeding as an important cause of in-hospital death, with a surprisingly high event rate of 10 % despite our policy of performing aneurysm repair as quickly as possible [29]. As we have reported previously, 73 % of in-hospital rebleeding events in our patient population occurred within 72 h of the index hemorrhage precipitating admission; the main risk factors for rebleeding were poor Hunt-Hess grade and large aneurysm size [29]. Non-modifiable admission predictors of mortality included age, pre-existing hypertension, and large aneurysm size, confirming the results of many other studies [10, 12, 14, 15, 30, 31].

Medical complications directly accounted for 15 % of in-hospital deaths in our study, which is lower than the 23 % proportion of deaths attributed to medical complications in the Cooperative Aneurysm Study [32]. This discrepancy may be explained by the fact that poor-grade patients were under-represented in the Cooperative Study. We previously reported in a subset of patients (*N* = 580) included our study cohort that fever of more than 38.3 °C, hyperglycemia of more than 200 mg/dl, and anemia treated with transfusion were significant predictors of either death or moderate-to-severe disability 3 months after SAH [10]. We did not find an independent association between these complications and in-hospital mortality in the present analysis, despite confirming that these long-term associations with poor functional outcome persist (unpublished data). This suggests that prolonged fever, hyperglycemia, and anemia contribute more directly to disability than to mortality *per se*, perhaps by contributing to neurological dysfunction and deconditioning.

Complications associated with refractory cerebral edema and cardiopulmonary dysfunction were both broadly associated with in-hospital death. In addition to global cerebral edema (which affected 26 % overall), ICP crisis or neurological worsening treated with bolus osmotherapy (22 %), hypernatremia (21 %), and clinical signs of brain stem herniation (14 %) were all associated with mortality after admission predictors were controlled for (Table 4). The association with hypernatremia primarily reflects the effect of osmotherapy since diabetes insipidus was formally diagnosed in only 4 % of our study population [10]. In our view, ICP, herniation, and brain edema complications generally constitute a downstream effect of severe diffuse early brain injury. Specific cardiopulmonary complications associated with mortality included hypotension of less than 90 mm Hg treated with pressors (which affected 24 % overall), congestive heart failure (8 %), and non-neurogenic myocardial ischemia (7 %), which was defined as acute or delayed troponin elevation possibly or probably due to coronary ischemia. The association of troponin elevation with stunned myocardium, hypotension, pulmonary edema, and poor outcome after SAH is well described [11, 33, 34] and supports multimodality monitoring data implicating hypoxia and hypotension as important causes of secondary brain injury [35].

A robust association between vasospasm and in-hospital mortality is notably absent in the present study. DCI from vasospasm was the adjudicated primary cause of death in 5 % of the cases, and DCI analyzed as a complication failed to independently predict death after admission risk factors were taken into account. By contrast, symptomatic vasospasm was the leading primary cause of death or disability among patients receiving treatment in the 1990 Cooperative Aneurysm Study, accounting for 28 % of all deaths [9]. This change most likely reflects the impact of interventions that have since reduced the likelihood of devastating brain infarction leading to withdrawal of support, including deployment of pharmacologic hemodynamic augmentation and intra-arterial therapy at the first sign of symptomatic vasospasm [8]. Even with aggressive management, there is ample evidence that brain infarction from DCI continues to be an important cause of cognitive impairment and disability after SAH [36, 37].

This study has several important limitations. First, these data were collected from a single highly specialized center and this may limit generalizability. Second, our overall management approach evolved over the 12-year enrollment period, as we eventually moved toward routinely offering of a 1-week trial of surgical or endovascular aneurysm repair and aggressive ICU support for all but the most moribund cases. Our attempt to identify an adjudicated “primary cause of death or neurological devastation leading to withdrawal of support” doubtlessly resulted in over-simplification in some cases, but we felt that trying to assign weights to multiple contributing factors would be even more arbitrary. Although we used pre-defined criteria for identifying medical complications, varying application of these criteria and observer bias by study team members that changed over the years may have affected the accuracy and consistency of how various findings and events were coded. We identified temporal trends showing lower mortality in Hunt-Hess grade 4 and 5 patients over time. In future studies, we will attempt to elucidate possible explanations for these improvements in outcome. Finally, as a consequence of restricting the scope of our analysis to in-hospital mortality, we did not analyze long-term survival or functional and quality-of-life outcomes after discharge.

## Conclusions

Our findings suggest that strategies directed toward minimizing early brain injury and aneurysm rebleeding, along with prevention and treatment of cardiovascular complications, hold the best promise for further reducing mortality after SAH. Neuroprotection directed at minimizing the initial catastrophic diffuse brain insult and interventions designed to stabilize acute physiological derangements are promising targets for therapy [8]. Early use of brain multimodality monitoring may aid in the development of strategies directed at minimizing metabolic crisis, brain tissue hypoxia, spreading depression, and other potential mediators of early brain injury [13, 35]. Routine use of anti-fibrinolytic therapy within 72 h of onset to minimize the risk of early rebleeding has been shown to be effective in a clinical trial and a single-center implementation study [38, 39]. Trials of acute sympatholytic therapy and advanced cardiovascular monitoring aimed at minimizing myocardial catecholamine toxicity and the frequency and severity of major adverse cardiovascular events deserve further study.

Premature withholding or withdrawal of support on the basis of self-fulfilling prophecies without offering a genuine trial of aggressive early resuscitation may be a powerful determinant of mortality after SAH [23]. We feel that, whenever possible, an initial trial of full support, at a minimum including aneurysm repair and ventriculostomy placement, should be offered to all potentially viable poor-grade SAH patients if consistent with their previously stated wishes. We have previously noted that an early aggressive trial of ICU support may actually improve satisfaction and subsequently make it easier for families to change the goal of care to comfort, knowing full well that everything possible was done [8, 40]. Finally, our findings provide a benchmark for evaluating SAH treatment centers and demonstrate that reductions in mortality from this devastating disease are possible.

## Key messages

- In-hospital mortality in this single-center cohort study of patients with SAH was 18 %
- 42 % died of brain death, 50 % were DNR, and only 8 % died despite full medical support
- Direct effects of severe hemorrhage, rebleeding, and medical complications were the most common causes of death
- Delayed cerebral ischemia from vasospasm did not predict mortality

## Additional file

Additional file 1:
**Methods Supplement.** This provides details regarding inclusion and exclusion criteria, aneurysm management, and a description of our intensive care unit management protocol and how it evolved over time. (DOCX 26 kb)

## Abbreviations

APACHE: Acute Physiology and Chronic Health Evaluation
CT: Computed tomography
DCI: Delayed cerebral ischemia
DNR: Do not resuscitate
GCS: Glasgow Coma Scale
ICP: Intracranial pressure
ICU: Intensive care unit
IQR: interquartile range
SAH: Subarachnoid hemorrhage
