# Supplementary material for: Subarachnoid hemorrhage: who dies, and why?
Source: Crit Care. 2015 Aug 31;19(1):309. doi: 10.1186/s13054-015-1036-0 (PMC4556224; doi:10.1186/s13054-015-1036-0)
Supplement: Additional file 1: — Methods Supplement. This provides details regarding inclusion and exclusion criteria, aneurysm management, and a description of our intensive care unit management protocol and how it evolved over time. (DOCX 26 kb) [file 13054_2015_1036_MOESM1_ESM.docx]

**Additional File 1: METHODS SUPPLEMENT**

Lantigua et al, “Subarachnoid Hemorrhage: Who Dies, and Why?”

*Inclusion and exclusion Criteria:* The diagnosis of SAH was established by the admission CT scan or by the presence of red blood cells and xanthochromia in the cerebrospinal fluid. Inclusion criteria included patients 18 years or older with aneurysmal or spontaneous nonaneurysmal SAH admitted with 14 days of the proximal bleeding event. Patients with SAH due to trauma, arteriovenous malformation rupture, hemorrhagic infarction, vasculitis, or other structural lesions were excluded.

*Aneurysm Management.* Clipping versus coiling of aneurysms was determined by the consensus of the neurosurgical team. Over the 12 year study period there was no major change in the proportion of patients treated with clipping versus coiling. Starting in 2000, we adopted a policy of offering aneurysm repair and a one-week trial of critical care to all but the most moribund grade 5 patients (e.g. brainstem failure).

*Details of Clinical Management.* All patients received nimodipine every 4 hours, phenytoin or levatiracetam perioperatively for seizure prophylaxis (predominantly levatiracetam as of 2008), and 0.9% normal saline at a rate of 1 mL/kg per hour with supplemental 5% albumin or normal saline to maintain central venous pressure >5 mm Hg. Staring in 2006, we discontinued the routine use of supplemental 5% albumin, and in 2008 we abandoned the routine use of CVP targets for fluid administration, focusing instead on maintaining even cumulative fluid balance, ultrasound markers of fluid responsiveness, and PICCO monitoring of global end diastolic volume in poor grade patients. External ventricular drainage (EVD) was placed in patients with symptomatic hydrocephalus or intraventricular hemorrhage with reduced level of consciousness. Starting in 2003, we adopted a policy of administering epsilon aminocaproic acid (4g, followed by 1 g/hr until angiography) to all patients at the time of diagnosis. All patients underwent digital subtraction angiography on admission, and serial transcranial Doppler sonography or CT angiography thereafter to monitor for vasospasm. Prior to 2003 we used SPECT scanning for mapping cerebral perfusion in selected patients, before switching to CT perfusion imaging. Standardized serial neurological examinations were performed off sedation throughout each day. Clinical deterioration from delayed cerebral ischemia (DCI) was treated with hypertensive hypervolemic therapy (HHT) to maintain systolic blood pressure (SBP) between 160-220 mm Hg, as required to reverse the neurological deficit. In 2006 we switched the first line vasopressor of choice from phenylephrine to norepinephrine, and switched our threshold for blood transfusion in the absence of DCI from 9 to 7 mg/dL of hemoglobin. When clinical evidence of DCI persisted for more than 2 hours despite HHT, cerebral angiography was used to identify vasospasm and balloon angioplasty or intra-arterial verapamil administration was performed whenever feasible. Head CT scans were performed on an as-needed basis for clinical purposes only; prearranged routine serial CT scans were not performed. cEEG monitoring for all cases of unexplained neuroworsening, and in all comatose poor grade patients, was performed routinely throughout the study period. Starting in 2006 we initiated a multimodality brain monitoring protocol for all poor grade (Hunt-Hess 4 and 5) patients, progressively adding brain tissue oxygen, microdialysis, Hemedex CBF, and EEG depth electrode monitoring to our standard montage between 2006 and 2009. MMM was used to detect worsening and trigger scanning and intervention in brain metabolic or electrical state, and to optimize CPP, ETCO2, temperature and sedation. We instituted hypothermia as a standard measure for medically-refractory ICP in 2007, and intra-arterial nicardipine as rescue therapy for refractory vasospasm in 2008.
